# Effects of Posture on Peripheral Circulation

**Published:** 1878-09

**Authors:** 


### The Effect of Posture on the Peripheral Circulation.

On the 18th of June last Mr. Lister read a paper on this subject before the Académie de Médicine. According to the report in "L'Union Médicale," he stated that he had been led to attend specially to the subject when studying the resection of the wrist for caries. In order to prevent the hæmorrhage he applied Petit's tourniquet upon the arm, after having raised the limb for some minutes. By this means the limb was rendered almost exsanguine. Later, in 1873, it occurred to him that this result was not the simple mechanical effect of gravitation, but was a reflex phenomenon caused by the emptying of the veins producing contraction of the muscular fibres of the arteries. In surgery this method of raising a limb, and then applying at its root a tourniquet, has all the advantages of the system of Esmarch without its inconveniences, such as the danger of forcing septic matters into the interstices of healthy tissues. In order to observe the effect better, Mr. Lister performed the following experiment on a horse. By means of cords and pulleys attached to the legs of a horse, he varied the position of one hind leg, at one time elevating it while the animal was on its back, at another time keeping the leg horizontal while the horse was on its side, at another time allowing it to stand upright with the leg downwards. The metacarpal artery having been exposed, it was seen that when the leg was raised the artery did not pulsate, and that the wound, being cleared of blood, resembled one made after death. By means of a gauge the diameter of the artery was ascertained. When the leg was raised the diameter of the vessel scarcely exceeded that of the same artery divided and emptied, while in the horizontal position, and especially while the limb was dependent, the enlargement of the vessel was considerable. By calculating the internal area from the external diameter, it was estimated that on changing the position from an elevated to a horizontal position, the calibre of the vessel was increased threefold, and that it became increased sixfold when the limb hung down.

Mr. Lister demonstrated the effect of this method on the arm of one of the servants of the Academy, and showed that if a limb was raised and a tourniquet applied it remained pale and bloodless, even when it was allowed to hang down, and on raising the limb again and removing the pressure, the color rapidly returned to it, in spite of its position, which was the same as that in which it had become pale and exsanguine before the application of the tourniquet. He explained this result by supposing that after the tissues of a limb have been deprived for a certain time of all circulation, there is, so to speak, a need for the circulation, and that this need acts as a stimulus, and determines a relaxation of the arteries by acting on the vaso-motor system just as warmth does. This stimulus of need of circulation, which causes the relaxation of the arteries, becomes stronger than the stimulus of relaxation of the veins excited by gravitation, which under other circumstances would have caused their contraction. in consequence, the reaction is strong in proportion to the duration of the constriction.

Another experiment consisted in exciting the circulation by a short run, then raising the arm for a few minutes, and then lowering it. The member became reddened and conjested just as after the application of cold. As evidence that these phenomena depend on a reflex action, Mr. Lister pointed out that if their cause were purely mechanical and physical, the lower part of the artery of a raised limb would have increased in size, since it would have been overfilled; but the actual state is the reverse. The femoral artery of the leg of a calf was exposed close to the abdomen. After the contraction caused by the irritation of the operation had ceased, he measured the external diameter of the vessel in different positions of the animal, and the results accorded exactly with his previous conclusions.

Finally, Mr. Lister pointed out the application of the theory to several phenomena, such as the good effects of the elevation of parts the seat of inflammation, and the treatment of epistaxis by elevation of the arm. Raising the arm produced, according to him, a reflex contraction of the arteries of the upper limbs, and, consequently, a sympathetic contraction of the facial arteries, leading to the cessation of the hæmorrhage.—*Lancet.*

